# Comparative Study of the Effect of Continuous Caudal Epidural With General Anesthesia Versus General Anesthesia on Intraoperative and Postoperative Analgesic Requirements for Lumbar Fixation

**DOI:** 10.1155/anrp/1582655

**Published:** 2026-05-14

**Authors:** Paula Michael El-Komos Samaan, Esraa Abdellatif Mohamed Shawky, Mahmoud Abd El-Aziz Ahmed Ghallab, Ahmed Mohamed El-Sayed El-Hennawy, Omar Mohamed Zafer Mohamed

**Affiliations:** ^1^ Faculty of Medicine, Ain Shams University, Cairo, Egypt, asu.edu.eg

**Keywords:** bupivacaine, caudal epidural, fluoroscopy, lumbar fixation, narcotics, pain scale, spine surgery

## Abstract

**Study design:**

The study was an interventional, randomized, prospective comparative clinical trial on 60 patients scheduled for lumbar fixation surgery.

**Study aim:**

The study goal was to justify the effectiveness of combined continuous caudal epidural with general anesthesia compared to general anesthesia alone as analgesia for lumbar fixation patients.

**Overview of Literature:**

Pain control during the intraoperative and postoperative period for spine surgeries is crucial. Much literature had proved that caudal analgesia is an acceptable alternative approach for intraoperative and postoperative analgesia.

Methods: 60 patients were equally allocated to two groups: Group A (Study group) and Group B (Control group). For Group A patients’, general anesthesia induction was followed by fluoroscopic‐guided caudal epidural catheter insertion. Thirty patients of Group B received general anesthesia only. Various parameters were assessed, including postoperative pain and hemodynamic parameters, time to rescue analgesia, estimated blood loss, surgeon satisfaction with the surgical field, total intraoperative and postoperative narcotics, and common postoperative complications of caudal epidural.

**Results:**

The intraoperative heart rate and mean blood pressure showed significant differences. Group A showed a significantly lower intraoperative fentanyl dose. Group A patients had nonsignificant lower blood loss and higher surgeon satisfaction with the surgical field. Postoperative numeric pain scale was significantly lower in the study group. Study group had significant less frequent postoperative morphine need and significant lower total 6‐h morphine dose. The rate of requesting the morphine first rescue dose was significantly later and lower in the study group. There was a significant less frequent sedation, in addition to a significant less frequent nausea and vomiting in the study group.

**Conclusions:**

Combined continuous caudal epidural with general anesthesia had lower intraoperative fentanyl dose and postoperative morphine dose, with overall lower opioid requirements and better pain control.

**Trial Registration:** Clinical trial.gov.identifier; NCT06929611

## 1. Introduction

### 1.1. Background

Pain control during the intraoperative and postoperative period for spine surgeries is crucial, as inadequate pain control can cause complications as severe as mortality [1].

Whereas general anesthesia is the most widely used approach in lumbar spine surgeries, the use of regional and neuraxial anesthesia is a newly rising analgesic approach [2]. Multimodal analgesia, with opioids, is administered for pain control during spine surgeries. Common opioid complications include respiratory depression, nausea, delirium, itching, constipation, and ileus [3].

The caudal epidural was initially applied as a blind anatomical technique. 68%–75% success rate of the “blind” approach to caudal epidural seems low in experienced hands; however, the use of a fluoroscopic technique for needle and catheter placement is a good tool for trying to achieve 100% success. Even with confirmation of the placement of the catheter, there can be migration of the catheter, including into the dural sac [4].

Caudal analgesia is an acceptable alternative approach for improved intra‐ and postoperative analgesia. A single injection caudal block has a relatively brief duration, so the epidural catheter continuous injection is performed to extend its duration as a continuous caudal epidural [5].

Nevertheless, caudal epidural anesthesia is an inadequately utilized analgesic approach, even a proven technique in providing analgesia and managing postoperative pain for lumbar spine procedures. Pre‐emptive caudal epidural works by blocking sensory stimuli at the spinal cord level. It has a prominent role in providing good pain control during and after lumbar spine surgeries [6].

Common adverse effects of caudal anesthesia include intravascular, subdural, or intraosseous injection, hypotension, infection, sacral osteomyelitis, nerve roots injury, hematoma formation, rectum perforation, urinary retention, local anesthetic toxicity, and delayed respiratory depression [7].

Studies have shown that caudal epidural has better postoperative analgesia than intravenous narcotics [8], and this clinical trial study provided further support for the evolving practice of combined continuous caudal epidural with general anesthesia in adults undergoing lumbar fixation. Furthermore, we determined the analgesic requirement, rescue analgesia, hemodynamic changes, and possible complications with continuous caudal epidural analgesia.

### 1.2. Study Aim

This study aim was to evaluate the effectiveness of combined continuous caudal epidural with general anesthesia compared to general anesthesia alone as analgesia for lumbar fixation patients, by recording both intraoperative and postoperative analgesic requirements, heart rate, blood pressure, and postoperative patient‐reported pain on the numeric pain scale.

### 1.3. Primary Outcome

- Determining the total amount of fentanyl that was consumed as intraoperative analgesia.

### 1.4. Secondary Outcome

- Determining the total amount of morphine that was given as analgesia for the first postoperative 6 h.
- Analyzing the numeric pain scale for the first 6 h postoperatively.
- Detecting intraoperative hemodynamic changes with continuous caudal epidural analgesia.
- Identifying the time interval to the first need for rescue analgesia.
- Detecting the intraoperative blood loss, surgeon satisfaction, and postoperative complication of caudal epidural.
- Justifying the effectiveness of continuous caudal epidural for analgesia in lumbar fixation surgeries.

## 2. Materials and Methods

### 2.1. Study Design

Study type was interventional, randomized, prospective comparative clinical trial. The study was conducted for six months, from January 2024 to June 2024, in Ain Shams University Hospitals’ operating theaters, following the approval of the Medical Research Ethical Committee under reference: FWA 000017585 (June 6, 2023).

### 2.2. Allocation and Randomization

Patients were randomly allocated using computer‐generated randomization and single‐blind technique (participant patients were blinded of group allocation, while the operators were not blinded) into two equal groups, Group A and Group B.

Group A: Patients undergoing lumbar fixation under combined continuous caudal epidural and general anesthesia.

Group B: Patients undergoing lumbar fixation under general anesthesia (opioid analgesia).

### 2.3. Sample Size Justification

Using the PASS 15 program for sample size calculation [9], the expected mean intraoperative narcotic consumption among study groups was 186.5 ± 21.6 μg and 106 ± 16.4 μg.

A sample size of 60 patients (30 patients for each group) can detect the difference between two groups with power > 90% and alpha error of 0.05.

### 2.4. Ethical Considerations

The study protocol had excellent ethical (approval from Medical Research Ethical Committee and informed consent), environmental (use of isoflurane and air), and economic considerations.

### 2.5. Participants

#### 2.5.1. Inclusion Criteria

##### 2.5.1.1. Age: Adult Patients From the Age of 21 Years–60 Years

- Sex: Both male and female.
- “American Society of Anesthesiologists” (ASA) Classification: patients with ASA Classification I and II.
- Level of surgery: lumber.
- Elective lumbar fixation surgeries.

#### 2.5.2. Exclusion Criteria

- Patients refusing to give informed consent.
- Patients who are less than 21 or more than 60.
- History of allergy to bupivacaine.
- History of opioid use.
- Emergency surgeries.
- Previous history of spine surgeries of any cause.
- Caudal injection site infection.
- Coagulopathy (genetic, acquired, and induced).
- ASA Classification: ASA III and IV.
- Uncorrected severe anemia or hypovolemia.
- Increased intracranial tension.

### 2.6. Study Procedures

#### 2.6.1. Preoperative Setting

**FIGURE 1:**
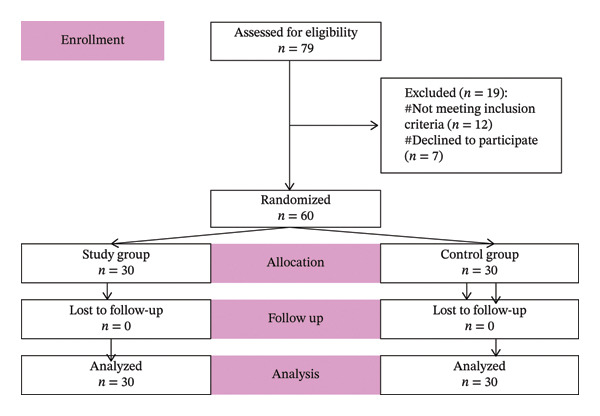
Study flow diagram (CONSORT).

A total of 79 patients were initially assessed for eligibility, of whom 19 were excluded, where 12 patients did not meet inclusion criteria, and 7 patients declined to participate (Figure 1: consort diagram). Randomization and a single‐blind technique were applied to 60 patients, using computer programs, where all patients of both groups consented to a combined continuous caudal epidural technique. Preoperative assessment included detailed history taking (including history of opioid use), thorough physical examination, radiological and laboratory investigations (including complete blood count [CBC], liver function test [LFT], kidney function test [KFT], partial thromboplastin time [PTT], and prothrombin time [PT]). All patients were fasting for 8 h preoperative. All patients were enlightened about study design, techniques, and objectives. All patients signed written informed consent before allocation. Patients were educated about the numeric pain scale, where 0 meant no pain at all and 10 meant intractable pain.

#### 2.6.2. Intraoperative Setting

Standard intraoperative monitoring was used, including electrocardiogram, noninvasive blood pressure measurement, pulse oximetry, end‐tidal CO_2_ measurement, and inhaled volatile agent concentration. Baseline parameters, including heart rate, oxygen saturation, systolic, diastolic, and mean blood pressure, were monitored. An intravenous line was placed. Induction of general anesthesia for patients of both groups was achieved using intravenous midazolam 0.04 mg/kg, fentanyl 1 μg/kg, propofol 2 mg/kg, and atracurium 0.5 mg/kg, and then endotracheal intubation and mechanical ventilation. General anesthesia maintenance was done using isoflurane 1.5% in oxygen and air (50:50) and atracurium 0.1 mg/kg every 20 min, maintaining end tidal CO_2_ between 35 and 40 mm Hg. 0.5 μg/kg fentanyl to be given with any increase in mean arterial blood pressure or heart rate more than 20% of baseline. The following factors were recorded for both groups: intraoperative heart rate and mean blood pressure, total intraoperative analgesia, estimated blood loss, and intraoperative surgeon satisfaction with the surgical field.

##### 2.6.2.1. Group A: (Continuous Caudal Epidural With General Anesthesia “Study Group”)

**FIGURE 2:**
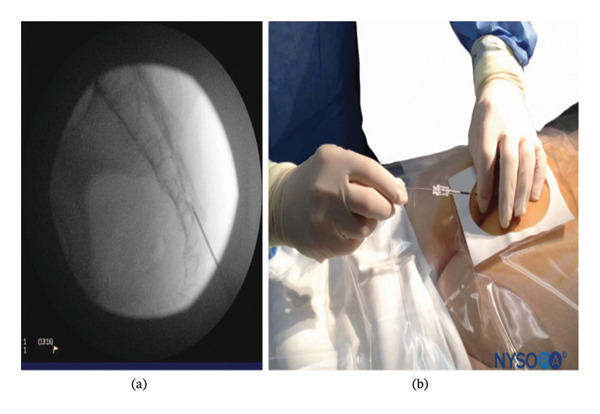
Lateral fluoroscopic image showing the 18‐gauge Tuohy needle correctly seated in the caudal epidural space.

**FIGURE 3:**
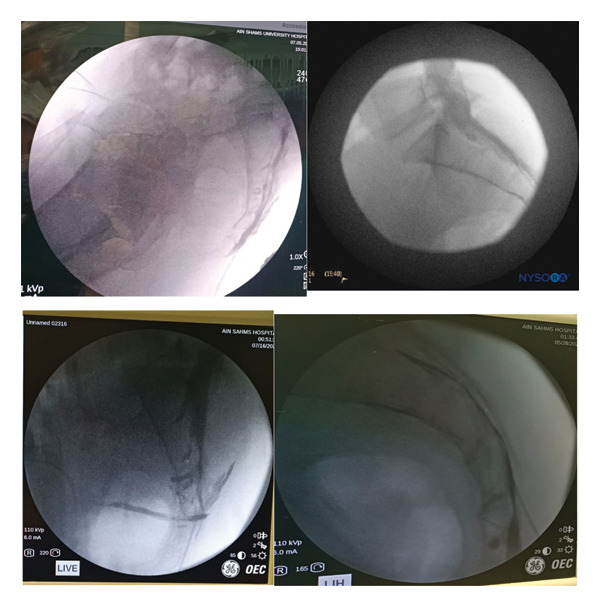
Lateral fluoroscopic image showing radiopaque contrast medium in the caudal and lower lumbar epidural spaces.

**FIGURE 4:**
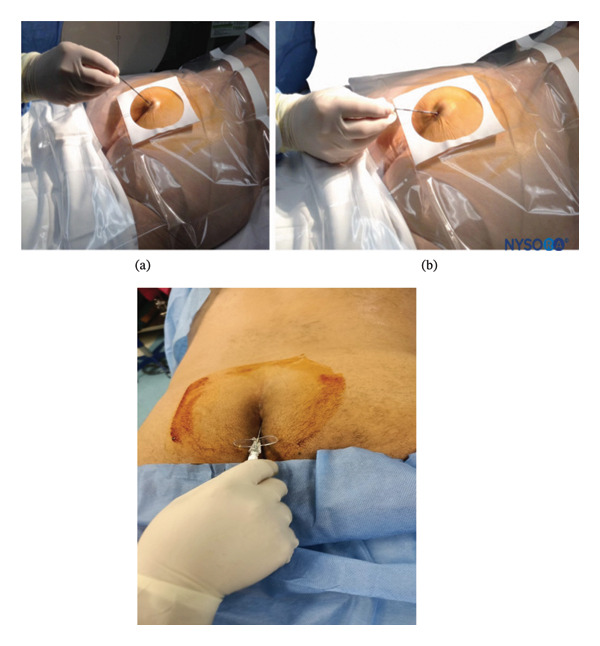
An 18‐gauge, Tuohy‐type needle is advanced from the skin into the sacral hiatus through the sacrococcygeal ligament.

**FIGURE 5:**
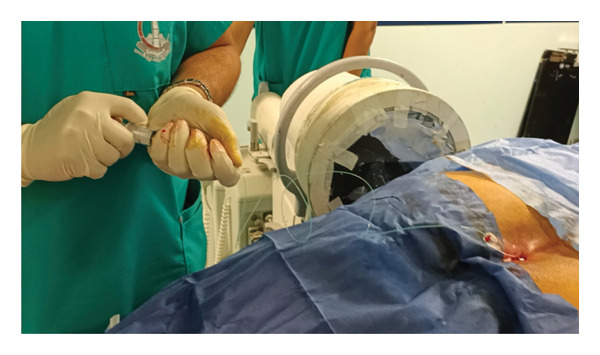
A continuous catheter with a stylet in place is being advanced through the 18‐gauge Tuohy needle placed in the canal.

**FIGURE 6:**
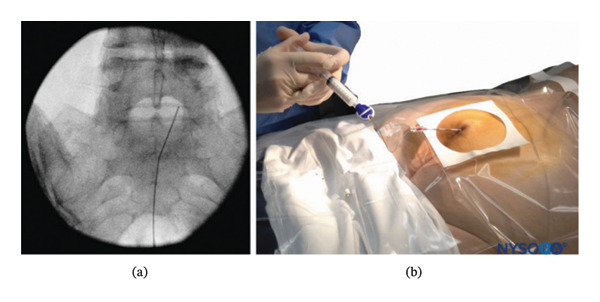
Anteroposterior fluoroscopic image showing the catheter advanced to the L5–S1 interspace.

**FIGURE 7:**
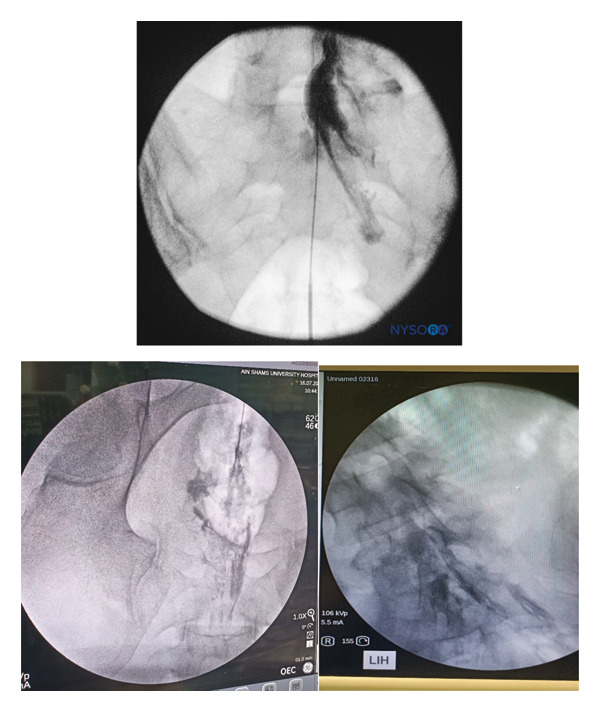
Anteroposterior fluoroscopic image showing catheter and radiopaque contrast medium in the epidural space.

After general anesthesia induction, patients were placed in the prone position for caudal epidural analgesia. Skin sterilization and surgical draping of the lower back area were done. Fluoroscopy (OEC Elite C‐arm) was utilized, and a lateral view was obtained to demonstrate the anatomical landmarks of the sacral canal (Figures 2 and 3). Using fluoroscopy, the caudal canal appeared as a translucent layer posterior to the sacral segments. The sacral hiatus was seen as a radiolucent opening at the base of the caudal canal. An 18‐gauge Tuohy‐type needle (Egemen International epidural set) was inserted in the midline into the caudal canal (Figure 4). A slight snap was sometimes felt when the needle pierced the sacrococcygeal ligament. Once the needle reached the ventral wall of the sacral canal, it was withdrawn and reoriented, directing it more cranially to advance further into the canal. Once the correct placement of the needle was confirmed using contrast dye (Omnipaque), the catheter was placed into the desired position and depth at the upper end plate of S1 (Figure 5), which were fluoroscopically confirmed as a lumbar epidural catheter via caudal route. The anteroposterior view was used once the epidural needle was safely placed within the canal, and the epidural catheter was advanced cephalad to L1‐L2 level (Figures 6 and 7). In this view, the intermediate sacral crests were visualized as opaque vertical lines on both sides of the midline. The position of the catheter was confirmed using contrast dye. The sacral foramina appeared as translucent circular areas lateral to the intermediate sacral crests. Dural sac usually (but not always) ends at S2, so before the local anesthetic was injected, cautious aspiration was done to exclude an unintentional intravascular or intrathecal needle position. Aspiration from the catheter should always occur for all injections, especially in a prone patient undergoing surgery, with exclusion of patients in case of CSF aspiration denoting dural puncture. A loading dose of 20 mL of 0.25% bupivacaine was given in the caudal canal to perform a sensory block and spare motor power. Then, a dose of 10 mL of 0.25% epidural catheter was given every 1 h intraoperatively, at 0 h and 1 h postoperatively. Finally, the catheter through which the bupivacaine was administered was removed 1 hour postoperatively.

##### 2.6.2.2. Group B: (General Anesthesia “Control Group”)

General anesthesia was induced as described above with the administration of extra doses of fentanyl as needed according to hemodynamic changes suggesting pain sensation.

#### 2.6.3. Postoperative Setting

At the end, patients were placed in supine position, and when patients became suitable for extubation, with stable hemodynamics and adequate muscle power, maintaining head support as a good clinical sign for adequate reversal, the neuromuscular blockade reversal was done by administration of neostigmine 0.05 mg/kg and atropine 0.01 mg/kg, thorough oral and endotracheal suction which was followed by extubation. Patients were nursed in the postanesthesia care unit (PACU) for recovery and monitoring of postoperative numeric pain scale at 0 h and management accordingly. Patients’ postoperative pain was followed up at the ward at 0, 1, 2, 4, and 6 h. Patients underwent close monitoring for the first 6 h after caudal injection for overdose or adverse reactions.

The following factors were assessed: postoperative pain assessment according to numeric pain scale 1–10 (0 = no pain, 10 = intractable pain) and hemodynamic parameters at 0, 1, 2, 4, 6 h, time to first need of rescue analgesia when the numeric pain scale was 3 or higher. Patients with a numeric pain scale ≥ 3 at any point in time received 0.05 mg/kg intravenous morphine (5 mg maximum), and total postoperative analgesia was provided in both groups. Common complications of caudal epidural in the postoperative period, including hypotension (mean blood pressure < 60 mmHg), bradycardia (heart rate < 50 beats/min), and urinary retention, were recorded and managed accordingly. Postoperative bradycardia, hypotension, and urinary retention were successfully managed using 10 mg of ephedrine, 0.5 mg of atropine, and a urine catheter, respectively.

### 2.7. Statistical Analysis

The collected data were coded, tabulated, and statistically analyzed using IBM Statistical Package for Social Sciences (SPSS) software Version 28.0, IBM Corp., Chicago, USA, 2021. Quantitative data were tested for normality using the Shapiro–Wilk test, then described as mean ± SD (standard deviation), minimum and maximum of the range, and then compared using an independent *t*‐test. Qualitative data were expressed as numbers and percentages and then compared using chi‐square test and Fisher’s exact test. Log‐rank test was used to compare the rate of requesting postoperative morphine. The level of significance was set at *p* value < 0.050, and otherwise was considered nonsignificant.

## 3. Results

A total of 79 patients were initially assessed for eligibility, of whom 19 were excluded, where 12 patients did not meet inclusion criteria, and 7 patients declined to participate (Figure 1: consort diagram). The average age of the patients was 47.7 years for Group A and 45.8 years for Group B, with 27 male patients and 33 female patients. There was a nonsignificant difference regarding patient demographic data, including age, gender, BMI, ASA grade, vertebrae fixation levels, and operation duration (Table 1).

**TABLE 1:** Demographic characteristics between the study groups.

Variables		Study group (total = 30)	Control group (total = 30)	*p* value
Age (years)		47.7 ± 8.1	45.8 ± 8.3	^^^0.391
Gender	Male	14 (46.7%)	13 (43.3%)	^#^0.795
	Female	16 (53.3%)	17 (56.7%)	

BMI (kg/m2)		28.8 ± 2.1	29.5 ± 2.1	^^^0.193
ASA	I	16 (53.3%)	14 (46.7%)	^#^0.606
	II	14 (46.7%)	16 (53.3%)	

Vertebrae fixation levels	L2‐L3	10 (33.3%)	8 (26.7%)	^#^0.573
	L3‐L4	17 (56.7%)	17 (56.7%)	^#^0.999
	L4‐L5	17 (56.7%)	18 (60.0%)	^#^0.793
	L5‐S1	8 (26.7%)	11 (36.7%)	^#^0.405

Number of vertebrae levels	One	12 (40.0%)	12 (40.0%)	^#^0.758
	Two	14 (46.7%)	12 (40.0%)	
	Three	4 (13.3%)	6 (20.0%)	

Operation duration (minutes)		138.5 ± 28.6	143.0 ± 25.2	^^^0.520

*Note:* Data are presented as Mean ± SD or number (%). ASA: “American Society of Anesthesiologists.” ^^^Independent *t*‐test. ^#^Chi‐square test.

Abbreviation: BMI = Body Mass Index.

**FIGURE 8:**
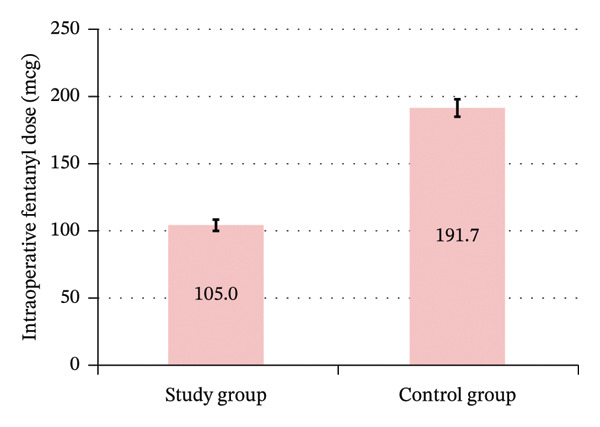
Intraoperative fentanyl dose between the study groups.

Group A had a significantly lower intraoperative fentanyl dose, with a mean of 105.0 ± 19.0 μg for Group A and 191.7 ± 35.6 μg for Group B (Table 2) (Figure 8).

**TABLE 2:** Intraoperative fentanyl dose, blood loss, and surgeon satisfaction with the surgical field between the study groups.

Variables	Study group (total = 30)	Control group (total = 30)	p value	Relative effect	
				Mean ± SE/Relative risk	95% CI
Fentanyl dose (mcg)	105.0 ± 19.0	191.7 ± 35.6	^< 0.001	−86.7 ± 7.4	−101.4–71.9
Blood loss (mL)	430.0 ± 140.6	513.3 ± 226.6	^0.092	−83.3 ± 48.7	−180.8–14.1
Surgeon satisfaction with the surgical field	18 (60.0%)	12 (40.0%)	§0.159	1.50	0.89–2.54
	11 (36.7%)	13 (43.3%)		Reference	
	1 (3.3%)	5 (16.7%)			

*Note:* Data are presented as Mean ± SD or number (%), unless mentioned otherwise. §Fisher’s exact test. ^Independent *t*‐test. Relative effect: Effect in the study group relative to that in the control group.

Abbreviations: CI = confidence interval; SE = standard error.

**FIGURE 9:**
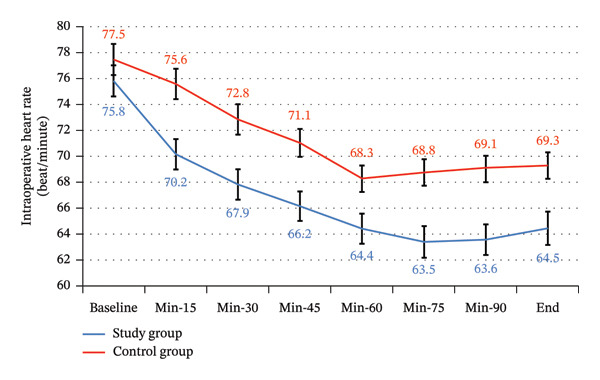
Intraoperative heart rate between the study groups.

**FIGURE 10:**
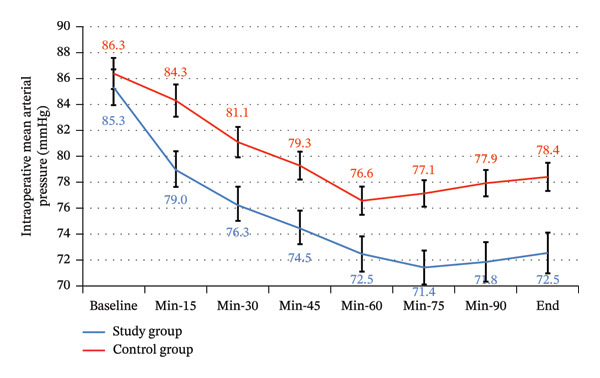
Intraoperative mean blood pressure between the study groups.

There was a statistically significant difference between the study and control groups for intraoperative heart rate and mean blood pressure throughout the intraoperative period (Table 3 and Table 4) (Figures 9 and 10).

**TABLE 3:** Intraoperative heart rate (beats/minute) between the study groups.

Intraoperative time	Study group (total = 30)	Control group (total = 30)	*p* value	Relative effect	
				Mean ± SD	95% CI
Baseline	75.8 ± 6.5	77.5 ± 6.5	0.323	−1.7 ± 1.7	−5.0–1.7
Min‐15	70.2 ± 6.3	75.6 ± 6.4	0.002	−5.4 ± 1.6	−8.6–2.1
Min‐30	67.9 ± 6.3	72.8 ± 5.9	0.003	−5.0 ± 1.6	−8.1–1.8
Min‐45	66.2 ± 6.4	71.1 ± 5.7	0.003	−4.9 ± 1.6	−8.0–1.7
Min‐60	64.4 ± 6.6	68.3 ± 5.5	0.016	−3.9 ± 1.6	−7.0–0.7
Min‐75	63.5 ± 6.4	68.8 ± 5.5	0.001	−5.3 ± 1.5	−8.4–2.3
Min‐90	63.6 ± 7.0	68.1 ± 5.4	0.008	−4.5 ± 1.6	−7.7–1.2
End	64.5 ± 7.5	69.3 ± 5.6	0.007	−2.8 ± 1.7	−8.3–1.4

*Note:* Data are presented as Mean ± SD, unless mentioned otherwise. ^^^Independent *t*‐test. Relative effect: Effect in the study group relative to that in the control group.

Abbreviations: CI = confidence interval; SE = standard error.

**TABLE 4:** Intraoperative mean blood pressure (mmHg) between the study groups.

Intraoperative time	Study group (Total = 30)	Control group (Total = 30)	*p* value	Relative effect	
				Mean ± SE	95% CI
Baseline	0.577	86.3 ± 6.6	85.3 ± 7.6	−1.0 ± 1.8	−4.7–2.7
Min‐15	0.006	84.3 ± 7.0	79.0 ± 7.4	−5.3 ± 1.9	−9.0–1.5
Min‐30	0.009	81.1 ± 6.5	76.3 ± 7.1	−4.8 ± 1.8	−8.3–1.3
Min‐45	0.008	79.3 ± 6.1	74.5 ± 7.3	−4.8 ± 1.7	−8.3–1.3
Min‐60	0.020	76.6 ± 5.9	72.5 ± 7.7	−4.2 ± 1.8	−7.7–0.7
Min‐75	0.002	77.1 ± 6.0	71.4 ± 7.4	−5.7 ± 1.7	−9.2–2.2
Min‐90	0.001	77.9 ± 5.6	71.8 ± 8.2	−6.1 ± 1.8	−9.7–2.5
End	0.003	78.4 ± 6.0	72.5 ± 8.7	−5.9 ± 1.9	−9.8–2.1

*Note:* Data are presented as Mean ± SD, unless mentioned otherwise. ^Independent *t*‐test. SE: standard error. Relative effect: Effect in the study group relative to that in the control group.

Abbreviation: CI = confidence interval.

**FIGURE 11:**
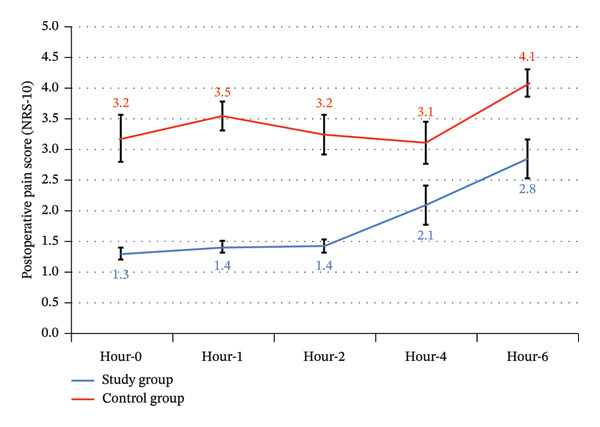
Postoperative pain score (NRS‐10) between the study groups.

Postoperative pain scale had significantly lower values in the study group from Hour 0 until Hour 6. Postoperative pain scale in the study group began at a low level and increased gradually until Hour 6. In contrast, in the control group, it started at a high level and showed a reduction followed by an elevation (Table 5) (Figure 11).

**TABLE 5:** Postoperative Numeric Pain Scale (NRS‐10) between the study groups.

Postoperative time	Study group (total = 30)	Control group (total = 30)	*p* value	Relative effect	
				Mean ± SE	95% CI
Hour‐0	1.3 ± 0.5	3.2 ± 2.1	< 0.001	−1.9 ± 0.4	−2.7–1.1
Hour‐1	1.4 ± 0.5	3.5 ± 1.2	< 0.001	−2.1 ± 0.2	−2.6–1.7
Hour‐2	1.4 ± 0.5	3.2 ± 1.8	< 0.001	−1.8 ± 0.3	−2.5–1.1
Hour‐4	2.1 ± 1.7	3.1 ± 1.9	0.037	−1.0 ± 0.5	−1.9–0.1
Hour‐6	2.8 ± 1.7	4.1 ± 1.2	0.002	−1.2 ± 0.4	−2.0–0.5

*Note:* Data are presented as Mean ± SD or number (%), unless mentioned otherwise. #Chi‐square test. ^Independent *t*‐test. Relative effect: Effect in the study group relative to that in the control group.

Abbreviations: CI = confidence interval; NA = not applicable; SE = standard error.

**FIGURE 12:**
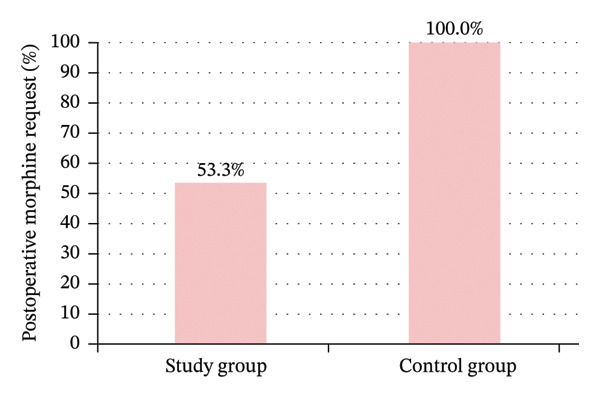
Postoperative morphine need between the study groups.

**FIGURE 13:**
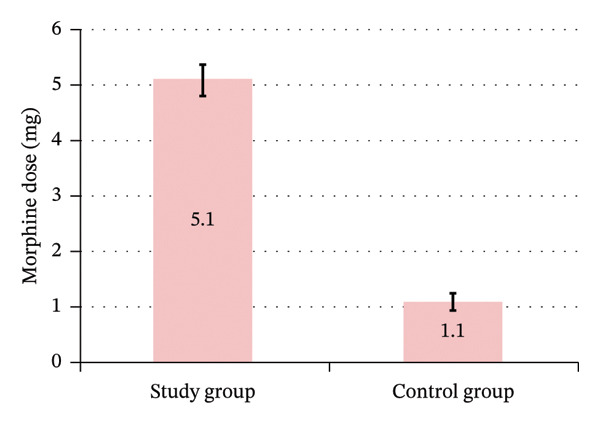
Postoperative time to morphine first rescue dose between the study groups.

**FIGURE 14:**
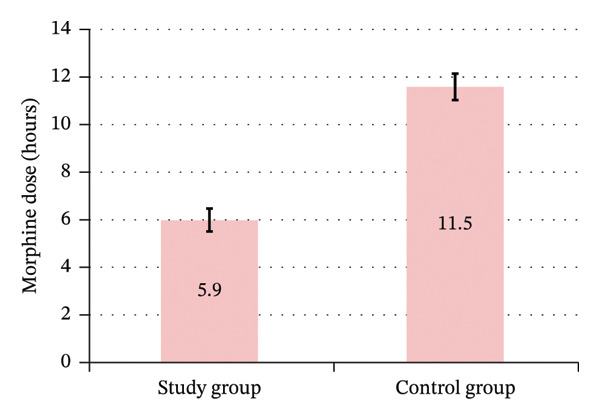
Postoperative total 6‐hour morphine dose between the study groups.

**FIGURE 15:**
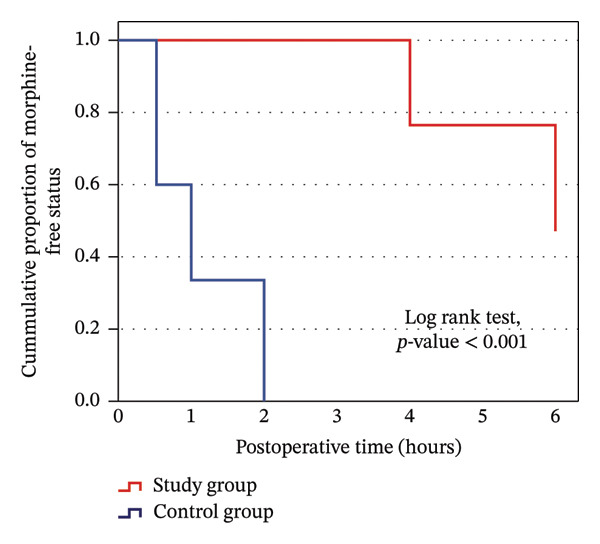
Kaplan–Meier curve for request of the first morphine dose among the study groups.

The study group had significant less frequent postoperative morphine need 53.3% (16 patients) of the study group versus 100% (all 30 patients) of the control group (Figure 12); among these, the study group had significantly longer time to morphine first rescue dose of 5.1 ± 1.0 h for Group A and 1.1 ± 0.7 h for hours for Group B, and a significantly lower total 6 h morphine dose of 5.9 ± 2.0 mg for Group A and 11.5 ± 3.0 mg for Group B (Figure 13), with significantly lower rate of requesting the first rescue analgesia dose (morphine) in the study group (Table 6) (Figures 14 and 15).

**TABLE 6:** Postoperative morphine requirement between the study groups.

Postoperative morphine requirement	Study group (Total = 30)	Control group (Total = 30)	*p* value	Relative effect	
				Mean ± SE	95% CI
Morphine request	16 (53.3%)	30 (100.0%)	^#^< 0.001	NA	
	Total = 16	Total = 30			
Time to morphine first rescue dose (hours)	5.1 ± 1.0	1.1 ± 0.7	^^^< 0.001	4.0 ± 0.2	3.5–4.5
Total 6‐h morphine dose (mg)	5.9 ± 2.0	11.5 ± 3.0	^^^< 0.001	−5.6 ± 0.8	−7.2–3.9

*Note:* Data are presented as Mean ± SD or number (%), unless mentioned otherwise. #Chi‐square test. ^Independent *t*‐test. Relative effect: Effect in the study group relative to that in the control group.

Abbreviations: CI = confidence interval; NA = not applicable; SE = standard error.

**FIGURE 16:**
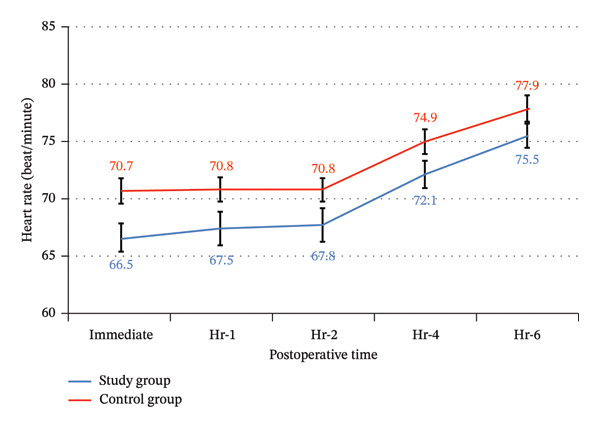
Postoperative heart rate among the study groups.

**FIGURE 17:**
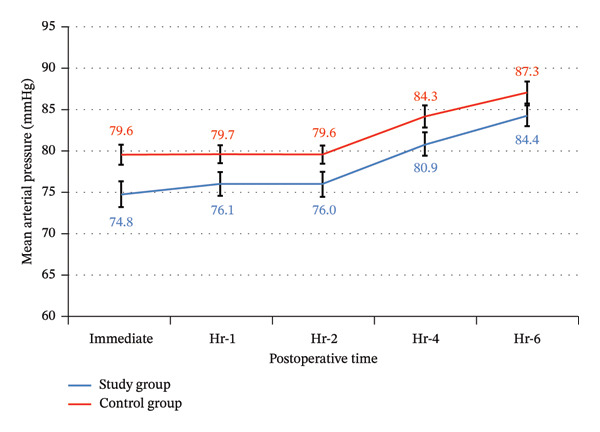
Postoperative mean arterial pressure among the study groups.

**FIGURE 18:**
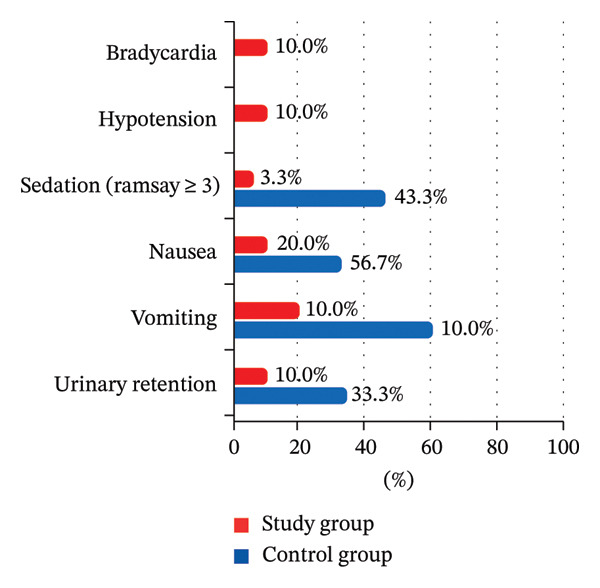
Postoperative side effects between the study groups.

There were no statistically significant differences between the study and control groups for blood loss and surgeon satisfaction with the surgical field (Table 2), postoperative heart rate, and mean blood pressure throughout the 6 postoperative hours, with significantly lower heart rate and mean blood pressure only immediately postoperative (Table 7) (Figures 16 and 17). There was more frequent bradycardia (heart rate < 50 beats/min), hypotension (mean blood pressure < 60 mmHg), and urinary retention. There was a significantly less frequent sedation (Ramsay ≥ 3), as well as a significantly less frequent postoperative nausea and vomiting in the study group (Table 8) (Figure 18).

**TABLE 7:** Postoperative heart rate and mean arterial pressure between the study groups.

**Time points**	**Study group (Total = 30)**	**Control group (Total = 30)**	**p** **value**	**Relative effect**	
				**Mean ± SE**	**95% CI**
*Heart rate (beat/minute)*					
PO immediate	70.7 ± 5.7	66.5 ± 7.6	0.018	−4.2 ± 1.7	−7.7–0.7
PO hr‐1	70.8 ± 5.7	67.5 ± 7.6	0.061	−3.3 ± 1.7	−6.8–0.2
PO hr‐2	70.8 ± 5.6	67.8 ± 7.3	0.079	−3.0 ± 1.7	−6.4–0.4
PO hr‐4	74.9 ± 6.1	72.1 ± 6.7	0.087	−2.9 ± 1.6	−6.2–0.4
PO hr‐6	77.9 ± 6.5	75.5 ± 6.3	0.144	−2.4 ± 1.6	−5.7–0.9

*Mean arterial pressure (mmHg)*					
PO immediate	74.8 ± 8.2	79.6 ± 6.2	0.014	−4.7 ± 1.9	−8.5–1.0
PO hr‐1	76.1 ± 8.2	79.7 ± 6.1	0.054	−3.7 ± 1.9	−7.4–0.1
PO hr‐2	76.0 ± 8.3	79.6 ± 6.1	0.060	−3.6 ± 1.9	−7.4–0.2
PO hr‐4	80.9 ± 7.4	84.3 ± 6.5	0.065	−3.4 ± 1.8	−7.0–0.2
PO hr‐6	84.4 ± 6.9	87.3 ± 7.3	0.129	−2.8 ± 1.8	−6.5–0.9

*Note:* Data are presented as Mean ± SD, unless mentioned otherwise. IO: Intraoperative. PO: postoperative. Relative effect: Effect in study group relative to that in control group.

Abbreviations: CI = confidence interval; SE = standard error.

**TABLE 8:** Postoperative side effects between the study groups.

Side effects	Study group (total = 30)	Control group (total = 30)	*p* value	Relative effect relative risk 95% CI
Bradycardia	3 (10.0%)	0 (0.0%)	§0.237	NA
Hypotension	3 (10.0%)	0 (0.0%)	§0.237	NA
Sedation (Ramsay ≥ 3)	1 (3.3%)	13 (43.3%)	#< 0.001	0.08 (0.01–0.55)
Nausea	6 (20.0%)	17 (56.7%)	#0.003	0.35 (0.16–0.77)
Vomiting	3 (10.0%)	10 (33.3%)	#0.028	0.30 (0.09–0.98)
Urinary retention	3 (10.0%)	0 (0.0%)	§0.237	NA

*Note:* Data are presented as numbers (%), unless mentioned otherwise. §Fisher’s exact test. #Chi‐square test. Relative effect: Effect in the study group relative to that in the control group.

Abbreviations: CI = confidence interval; NA = not applicable.

### 3.1. Discussion

Patients undergoing lumbar surgery experience postoperative severe pain, with a higher incidence of complications relating to this pain. Hence, there is a need for adequate early pain relief. The goal of pain control is to achieve patient comfort and satisfaction, aiming for rapid mobilization with a low incidence of deep vein thrombosis, fewer perioperative cardiac and pulmonary complications, and ensuring a cost‐effective, rapid recovery, and pain‐free rehabilitation [10].

Pain transmission leads to central nervous system plasticity, causing prolonged, enhanced perception of pain even postoperatively with no further painful stimuli. A single‐caudal epidural dose administrated 20 min before any noxious stimulus is an effective method for postoperative pain control. A preemptive caudal epidural loading dose injected prior to the surgery as well as further intraoperatively dosing is a safe and effective method to control postoperative pain. The epidural bupivacaine adheres to the nerve root in about 20 min. It blocks the sensation of a painful stimulus, preventing CNS neuroplasticity and causing adequate pain control during the first 24 postoperative hours. This results in decreasing both intraoperative and postoperative narcotics consumption with a lower pain scale. However, caudal epidural analgesia is an infrequently utilized analgesic technique for lumbosacral spine surgery, for which general anesthesia, without complementary neuraxial analgesia, is more commonly used. The caudal epidural effect on intraoperative and postoperative hemodynamics depends on the absorption rate of epidural bupivacaine, extent of the spread of spinal dermatomes, and sympathetic nervous system block [11].

A caudal epidural catheter (lumbar epidural catheter via caudal route) can permit continuous control up to higher levels of dermatomes. It does not interfere with surgical field, where a continuous caudal approach provided access to the epidural space, which was otherwise precluded by lumbar fixation surgery. Generally, caudal epidural is primarily a safe well‐documented route for providing analgesia with a high success rate for the fluoroscopic‐guided technique, with a few easily managed complications. Lumbar spine surgeries are done in the prone position after induction of general anesthesia, which was the same approach for the entry point caudal epidural needle, making it easy and of choice for analgesia, especially for lumbar fixation surgeries.

In our study, fluoroscopic‐guided continuous caudal epidural with an initial dose of 20 mL of 0.25% bupivacaine was injected in the caudal canal. Then, a dose of 10 mL of 0.25% bupivacaine was given through the epidural catheter every hour intraoperatively and at 0 and 1 h postoperative, along with general anesthesia for study group versus general anesthesia alone for the control group.

Our study demonstrated a significant lower intraoperative fentanyl dose among the study group of combined continuous caudal epidural with general anesthesia than that of the control group, ensuring better analgesia with continuous caudal epidural. This finding was consistent with a study, using a combined single‐injection caudal block with general anesthesia, which also showed a significant decrease in intraoperative narcotic with caudal epidural [9]. Another randomized clinical trial supported our finding, using a single dose of caudal epidural ropivacaine, which showed a significant lower intraoperative fentanyl dose and total requirement among the study group [12]. Furthermore, a randomized clinical trial, using caudal epidural ropivacaine, also showed significantly lower intraoperative fentanyl requirement for the study group than the control group with general anesthesia [11].

In this study, there were statistically significant differences between the study and control groups for intraoperative mean heart rate and mean blood pressure throughout the intraoperative period. Study group showed that 3 of 30 patients had postoperative bradycardia and hypotension in the caudal epidural group, which was adequately managed by 0.5 mg atropine and 10 mg ephedrine. This study’s results were consistent with a previous study’s findings showing a significant reduction in intraoperative hemodynamics [9]. However, these results differ from one that recorded no significant reduction in heart rate and blood pressure [11].

Our study using the numeric pain scale found significantly lower postoperative pain scores in the caudal epidural group. This aligns with other studies that used Visual Analogue Score [11, 13–15].

In our study, the caudal epidural group showed less frequent postoperative morphine need and experienced a significant delay in the first rescue analgesia. The average duration of the first need for rescue analgesia was recorded at 5.1 h for the caudal epidural group and 1.1 h for the control group, with a significantly lower total postoperative morphine dose of 5.9 mg for the caudal epidural group versus 11.5 mg for the control group (Table 6). This aligns with previous studies [9, 11–13].

While other investigators identified statistically significant lower surgical blood loss [9, 15], our results were similar but failed statistical significance.

Our study demonstrated significantly less frequent postoperative nausea and vomiting, as was reported in a previous investigation [9]. Postoperative bradycardia, hypotension, and urinary retention were infrequent and treated successfully.

### 3.2. Limitations and Future Research Directions

Time of mobilization was not one of the results recorded during the study, as our Neurosurgery Department’s protocol was to mobilize postsurgical patients after 8 h; hence, the effect of caudal epidural on early ambulation cannot be justified during our 6‐h study. We recommend further study regarding early mobilization and its advantages, and 24‐h postoperative study with catheter in place for long‐term pain control.

Sample size was relatively small and single‐center which may limit external validity. It was calculated by Ain Shams University community department upon similar studies, and a sample size of 60 patients was determined to provide significant data. We recommend further multicenter studies with bigger sample sizes.

## 4. Conclusion

The use of combined continuous caudal epidural with general anesthesia in lumbar fixation surgery safely lowered intraoperative fentanyl dose and postoperative morphine dose, with overall lower opioid requirements and better pain control.

## Funding

No external funding sources were used; this study was funded only by the authors using available Ain Shams University hospitals’ resources.

## Ethics Statement

This study received ethical approval from the Research Ethics Committee number (REC/IRB number): FWA 000017585.

## Conflicts of Interest

There authors declare no conflicts of interest.

## Data Availability

The data that support the findings of this study are available from the corresponding author upon reasonable request.
